# The lncRNA H19 promotes epithelial to mesenchymal transition by functioning as miRNA sponges in colorectal cancer

**DOI:** 10.18632/oncotarget.4154

**Published:** 2015-06-05

**Authors:** Wei-Cheng Liang, Wei-Ming Fu, Cheuk-Wa Wong, Yan Wang, Wei-Mao Wang, Guo-Xin Hu, Li Zhang, Li-Jia Xiao, David Chi-Cheong Wan, Jin-Fang Zhang, Mary Miu-Yee Waye

**Affiliations:** ^1^ School of Biomedical Sciences, The Chinese University of Hong Kong, Shatin, New Territories, Hong Kong, P.R. China; ^2^ Croucher Laboratory for Human Genomics, The Chinese University of Hong Kong, Shatin, New Territories, Hong Kong, P.R. China; ^3^ Guangzhou Institute of Advanced Technology, Chinese Academy of Sciences, Guangzhou, P.R. China; ^4^ Department of Infectious Diseases, Peking University Shenzhen Hospital, Shenzhen, P.R. China; ^5^ Department of Clinical Laboratory, Nanshan Affiliated Hospital of Guangdong Medical College, Shenzhen, P.R. China; ^6^ Department of Orthopaedics & Traumatology, The Chinese University of Hong Kong, Prince of Wales Hospital, Shatin, Hong Kong, P.R. China

**Keywords:** miRNA sponges, lncRNA, ceRNA

## Abstract

Recently, the long non-coding RNA (lncRNA) H19 has been identified as an oncogenic gene in multiple cancer types and elevated expression of H19 was tightly linked to tumorigenesis and cancer progression. However, the molecular basis for this observation has not been characterized in colorectal cancer (CRC) especially during epithelial to mesenchymal transition (EMT) progression. In our studies, H19 was characterized as a novel regulator of EMT in CRC. We found that H19 was highly expressed in mesenchymal-like cancer cells and primary CRC tissues. Stable expression of H19 significantly promotes EMT progression and accelerates *in vivo* and *in vitro* tumor growth. Furthermore, by using bioinformatics study and RNA immunoprecipitation combined with luciferase reporter assays, we demonstrated that H19 functioned as a competing endogenous RNA (ceRNA) for miR-138 and miR-200a, antagonized their functions and led to the de-repression of their endogenous targets Vimentin, ZEB1, and ZEB2, all of which were core marker genes for mesenchymal cells. Taken together, these observations imply that the lncRNA H19 modulated the expression of multiple genes involved in EMT by acting as a competing endogenous RNA, which may build up the missing link between the regulatory miRNA network and EMT progression.

## INTRODUCTION

Colorectal cancer is the third leading cause of cancer patient's death worldwide, with around 1.2 million new cancer cases each year [1]. Although encouraging progress in diagnosis and cancer therapy has been achieved in the past decade, the overall survival rate remains unfavorable. Growing incidence and poor outcome of CRC has intensified attempts towards unraveling the underlying pathological mechanisms of CRC progression. Recently, emerging evidence highlighted EMT as an integral component of CRC and EMT-related molecule may act as novel targets for clinical prognosis and therapy [2]. The epithelial to mesenchymal transition is a process characterized by loss of cell-cell adhesion and gain of migratory and invasive traits. The transdifferentiation from quiescent epithelial cells into motile mesenchymal cells is essential for embryogenesis, fibrosis, tissue repair, wound healing, and tumor progression [3, 4]. Under pathological conditions, the epithelial to mesenchymal transition occurs at the initial stage of cancer metastasis. For instance, the colorectal carcinoma cells located in the frontier invasive section tend to possess mesenchymal properties such as poor differentiation, high proliferation rate, loss of cell polarity as well as enhanced ability of migration and invasion [5]. A number of key transcription factors were identified to potentiate EMT progression such as TWIST, ZEB1, ZEB2, SNAIL1, and SNAIL2 [6, 7]. Not surprisingly, during epithelial to mesenchymal transition, to adopt enhanced migratory and invasive characteristics, epithelial cells must destroy the extracellular constraints, which consist of cell adhesion molecules such as E-cadherin and p120 [8, 9]. Meanwhile, the expression of mesenchymal markers Vimentin would increase and hence contribute to cell motility and adhesion [10].

The long non-coding RNA (lncRNA) H19 gene is located on chromosome 11 in human and is a maternally expressed imprinted gene that plays a vital role in mammalian development [11]. Although H19 has been intensively studied in genomic imprinting, however, the pathological function of H19 as a non-coding RNA is only recently being elucidated. Rising evidence showed that H19 was upregulated in diverse cancer types including CRC [12, 13], esophageal cancer [14], bladder cancer [15, 16], breast cancer [17, 18], and hepatocellular carcinoma [19, 20]. The overexpression of H19 in cancer patients highlighted its tumorigenic properties, while the detailed molecular mechanisms remain poorly understood.

MiRNAs belong to a cluster of functionally active non-coding RNAs and exert their function by annealing to target sites located in the mRNA transcripts, with which they form the RNA-induced silencing complex (RISC) and subsequently trigger mRNA degradation or translational inhibition [21–23]. It's well documented that aberrant miRNA expression profiles have been causally connected to epithelial to mesenchymal transition and tumor progression [24, 25]. Recently, the competing endogenous RNA (ceRNA) hypothesis proposes that a large number of non-coding RNA might function as molecular sponges for miRNAs and hence functionally liberate other RNA transcripts targeted by aforementioned active miRNAs [26]. Furthermore, several ceRNAs were subsequently identified by concomitant studies such as PTENP1 [27], CD44 3′UTR [28], Linc-MD1 [29], and HMGA2 [30], pinpointing the biological importance of ceRNA.

We hypothesized that lncRNA H19 contributes to mesenchymal phenotypes by regulating relevant signaling networks in epithelial cells. We found that H19 expression was increased in both chemotherapy- and TGF-β1-induced EMT models. Subsequent results demonstrated that overexpression of H19 functioned as ceRNA and promoted epithelial to mesenchymal transition, leading to de-repression of the mesenchymal marker genes modulated by H19-targeting miRNAs. Further experiments showed that H19 potentiated tumorigenicity both *in vitro* and *in vivo*. Finally, clinical studies presented upregulation of H19 in colon cancer tissues when compared with adjacent normal tissues. Taken together, this study pinpointed H19 as a positive regulator of epithelial to mesenchymal transition.

## RESULTS

### LncRNA H19 was increased in mesenchymal-like methotrexate resistant HT-29 cells

**Figure 1:**
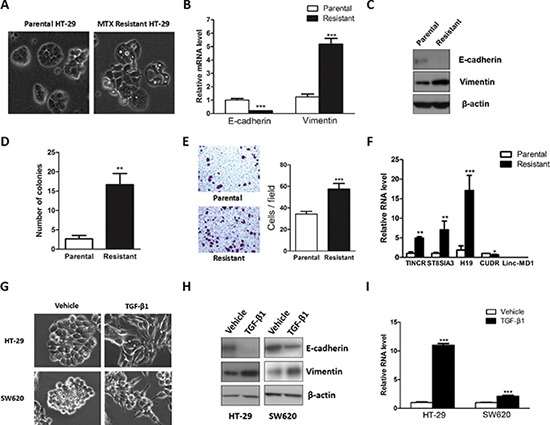
Upregulation of lncRNA H19 was observed in mesenchymal-like colon cancer cells **A.** Phase-contrast photomicrographs of the parental and Methotrexate resistant HT-29 cells. **B.** Relative mRNA levels of E-cadherin and Vimentin were monitored in the parental and Methotrexate resistant HT-29 cells. **C.** Protein levels of E-cadherin and Vimentin were evaluated in the parental and resistant HT-29 cells. **D.** The anchorage-independent growth capacity was determined by soft agar assay. **E.** The cell migration capacity was evaluated by transwell assay. **F.** The expression levels of candidate lncRNAs were monitored by qRT-PCR. **G.** HT-29 and SW620 cells were seeded into a 12 well plate and subsequently treated with TGF-β1 for 72 hours. Phase-contrast photomicrographs were captured. **H.** Protein expression levels of E-cadherin and Vimentin were monitored in HT-29 and SW620 cells to evaluate the effect of TGF-β1. **I.** qRT-PCR was performed to measure the expression level of H19 after TGF-β1 treatment. *P < 0.05; **P < 0.01; ***P < 0.001.

Emerging evidence implicates that cancer progression is tightly involved with EMT, which enables the cancer cells to acquire mesenchymal phenotype and metastasize towards distant sites. A strong association between drug resistance and the acquisition of EMT has been implicated in a burgeoning body of literature [31–33]. Intriguingly, several chemotherapeutic drugs may enrich cancer cells with mesenchymal phenotype through eliminating non-mesenchymal cells, which results in the presence of mesenchymal-like morphology and characteristics in resistant cancer cells [34, 35]. Methotrexate (MTX), an inhibitor of Dihydrofolate reductase (DHFR), exerts its anticancer function by competing for the active binding site in DHFR. It is well established that MTX plays a vital role in chemotherapy against a variety of human malignancies such as lymphoma, breast cancer, osteosarcoma, and colon cancer [36]. Hence, to gain insights into the functional role of lncRNA-mediated EMT progression, we first took advantage of chemotherapeutic drug-mediated EMT model MTX resistant HT-29 cells for relevant analysis [37]. In previous study, MTX resistant HT-29 cells displayed significant reduction of E-cadherin, an adherens junction protein essential for the extracellular structural architecture of epithelial cells, suggesting that MTX resistant cells may acquire mesenchymal property and undergo EMT [38]. To verify our hypothesis, we first observed the morphological changes under the microscope and remarkable elongated fibroblast-like shape was found when compared to the parallel parental HT-29 cells (Figure 1A). We subsequently monitored the mRNA and protein levels of two well-characterized marker genes E-cadherin and Vimentin, the hallmark of EMT. Significant downregulation of E-cadherin and concomitant upregulation of Vimentin were displayed in both mRNA and protein levels (Figure 1B and 1C). Following this observation, we further evaluated their migration and anchorage-independent growth capacity, which represents an *in vitro* characteristic of EMT in malignant cells. In accordance with precious results, MTX resistant HT-29 cells displayed enhanced colony formation and cell migration capacity (Figure 1D and 1E). To identify the putative lncRNAs involved in EMT progression, five previous reported lncRNAs were chosen and subjected to qRT-PCR to compare their expression profiling in this model [39]. All the candidate lncRNAs except Linc-MD1 presented significant dysregulation in MTX resistant HT- 29 cells (Figure 1F). Among the up-regulated lncRNAs, H19 displayed most dramatic change in the mRNA level and thus we chose H19 for further investigation.

To further characterize the different cell growth property in the parental and Methotrexate resistant HT-29 cells, we performed the cell proliferation related assays in our studies. Cell proliferation assay were performed and significant increased cell proliferation rate was observed in the Methotrexate resistant HT-29 cells (Supplementary Figure 1A and 1B). Subsequently, cell cycle progression was analyzed by flow cytometry. Compared with parental HT-29 cells, Methotrexate resistant HT-29 cells presented impaired G1 phase and increased G2/M phase (Supplementary Figure 1C and 1D). In order to further elucidate the different expression profiles of genes involved in G1/S phase transition, qRT-PCR was conducted and several promoter genes of G1/S phase transition were significantly upregulated (Supplementary Figure 1E).

### TGF-β1 potentiated H19 expression

To further assess the role of H19 in EMT, we used a well-characterized EMT inducer transforming growth factor-β1 (TGF-β1) to establish a canonical EMT model. Treatment with TGF-β1 resulted in the conversion from epithelial to the fibroblast-like feature in both HT-29 and SW620 cells (Figure 1G). As shown in Figure 1G, TGF-β1-treated epithelial cells displayed non-polarized and spindle-shaped morphology. Consistent with these morphology changes, a decrease of E-cadherin and increase of Vimentin was displayed in the protein level (Figure 1H), suggesting that the EMT model was successfully established. We subsequently monitored the expression level of H19 before and after treatment with TGF-β1. Consistent with a recent report [40], the qRT-PCR results revealed that H19 was dramatically upregulated after treatment with TGF-β1 (Figure 1I). Collectively, these finding supported that H19 may be involved in EMT progression.

### Overexpression of H19 promoted EMT progression

**Figure 2:**
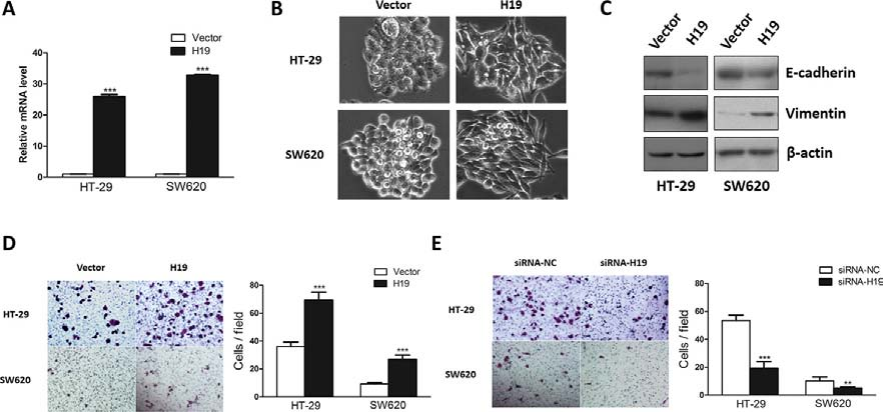
Ectopic expression of H19 promoted epithelial to mesenchymal transition **A.** H19 stable expression cell lines were generated by retrovirus infection. Relative H19 expression level was determined by qRT-PCR. **B.** The morphology of HT-29 and SW620 cells transduced with H19 or control vector were shown. **C.** The expression of EMT marker genes E-cadherin and Vimentin were evaluated in H19-overexpressing HT-29 and SW620 cells. **D.** The effect of ectopic H19 expression in cell migration was assessed by transwell assay. **E.** The effect of silencing H19 expression on cell motility was monitored by transwell assay. **P < 0.01; ***P < 0.001.

To elucidate the function of H19 in EMT, we performed the gain-of-function analysis using retroviral transduction of H19 in human colon cancer cells HT-29 and SW620. The overexpression of H19 was determined by qRT-PCR (Figure 2A) and semi-quantitative RT-PCR (Supplementary Figure 2). Overexpression of H19 resulted in morphological alteration and H19-overexpressing cells displayed elongated mesenchymal-like properties (Figure 2B), suggesting that these cells were undergoing epithelial to mesenchymal transition. Furthermore, in accordance with previous results, increased expression of H19 led to downregulation of epithelial marker E-cadherin and upregulation of mesenchymal marker Vimentin (Figure 2C), indicating that H19 might potentiate the transdifferentiation from epithelial cells to mesenchymal cells. As mesenchymal cells present enhanced migration ability, we next assessed the effect of overexpressing H19 in the regulation of cell migration capacity. The transwell assay showed that overexpression of H19 significantly promoted cell migration whereas inhibition of H19 suppressed cell migration in HT-29 and SW620 cells (Figure 2D and 2E).

### MiR-138 and miR-200a targeted H19

**Figure 3:**
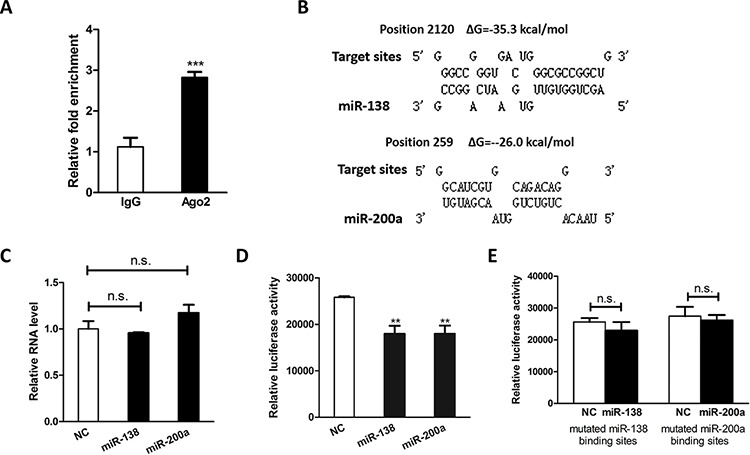
H19 functions as an miRNA decoy **A.** H19 RNA level in the immunoprecipitates were measured by qRT-PCR. **B.** Schematic diagrams of the mutual interactions between miRNA and H19. The calculated ΔG values are given to the top (kcal/mol). **C.** qRT-PCR results showed the relative RNA level of H19 after ectopic expression of miR-138 and miR-200a. **D.** HEK293 cells were transfected with mature miRNA mimics in combination with luciferase reporter harboring full length H19. The effects of miR-138 and miR-200 on the luciferase activity were determined by luciferase reporter assays. The activities of Firefly luciferase were normalized to the activities of β-Galactosidase expression vector. **E.** HEK293 cells were transfected with mature miRNA mimics in combination with luciferase reporter harboring mutated miRNA binding sites. The effects of miR-138 and miR-200 on the luciferase activity were determined by luciferase reporter assays. The activities of Firefly luciferase were normalized to the activities of β-Galactosidase expression vector. **P < 0.01.

Previous study showed that H19 associated with miRNA ribonucleoprotein complexes and served as a natural molecular sponge for let-7 family [41, 42]. Growing evidence indicated that lncRNAs may act as a decoy to sequester miRNAs and hence modulate their downstream targets. [27–30] Given that miR-138 and miR-200a are known to attenuate EMT [43–45], we posited that H19 might promote EMT by serving as a miRNA sponge and hijacking these two miRNAs. It is well defined that miRNA exerts its function by binding to Ago2, a core component of the RNA-induced silencing complex (RISC). To assess whether H19 associates with RISC complex, RNA immunoprecipitation (RIP) assay was performed by using antibodies against human Ago2. According to the result from RIP experiment, H19 was preferentially enriched in Ago2-containing beads compared to the beads harboring control immunoglobulin G (IgG) antibody (Figure 3A). On the other hand, bioinformatics tools revealed potential binding sites for miR-138 and miR-200a in H19 gene (Figure 3B). Subsequent qRT-PCR analysis showed overexpression of miR-138 and miR-200a did not affect H19 mRNA expression (Figure 3C). However, overexpression of miR-138 and miR-200a suppressed the luciferase activity of luciferase reporter harboring full length H19 (Figure 3D) while site-directed mutagenesis of these two binding sites successfully abolished the suppressive effects (Figure 3E), indicating miR-138 and miR-200a mediated translational suppression-like effect rather than RNA degradation. Collectively, these results pinpoint a role of H19 as a miRNA decoy for miR-138 and miR-200a.

### H19 modulated expression of endogenous miR-138 target Vimentin

**Figure 4:**
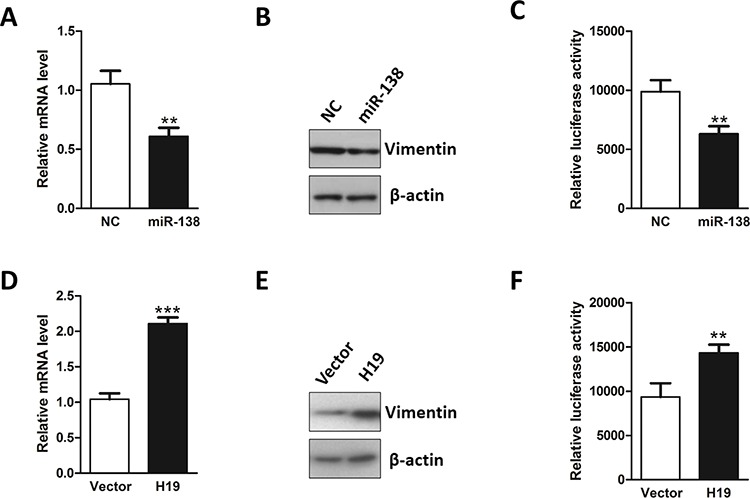
H19 functions as a sponge for Vimentin-targeting miRNA **A.** MiR-138 was transfected into HT-29 cells and the mRNA level of Vimentin was evaluated by qRT-PCR. **B.** After transfection with miR-138, the protein level of Vimentin was examined by western blot. **C.** Luciferase reporter assay was conducted to test the interaction between miR-138 and Vimentin mRNA using the luciferase reporter containing the coding region of Vimentin. **D.** The effect of H19 overexpression in the regulation of Vimentin mRNA level was monitored by qRT-PCR. **E.** The effect of H19 upregulation in the modulation of Vimentin protein level was monitored by western blot. **F.** Luciferase reporter assay was performed to examine the effect of H19 in antagonizing miR-138-mediated suppression on the coding region of Vimentin. **P < 0.01; ***P < 0.001.

Liu *et al*. demonstrated that miR-138 promoted EMT and cell migration through directly targeting mesenchymal marker gene Vimentin [45]. Consistent with these findings, ectopic expression of miR-138 significantly reduced the mRNA and protein level of Vimentin (Figure 4A and 4B). Further luciferase reporter assay showed that miR-138 inhibited luciferase activity of luciferase reporter harboring coding region of Vimentin gene (Figure 4C). To determine whether H19 affects Vimentin expression, we monitored both mRNA and protein levels of Vimentin in H19-overexpressing HT-29 cells. Significant increase in the mRNA and protein levels for Vimentin were observed in response to H19 overexpression (Figure 4D and 4E). Moreover, we also synthesized siRNA targeting H19 and monitored the effect of H19 in regulating Vimentin expression. Consistent results were achieved in our study (Supplementary Figure 3A and 3B). And we also found that stable expression of H19 restored miR-138-mediated suppression on luciferase activity (Figure 4F). Taken together, these data indicate that enhanced H19 expression inhibits miR-138 function, eliminating the repression on Vimentin induced by miR-138 and promoting epithelial to mesenchymal transition.

### H19 modulated expression of endogenous miR-200a targets ZEB1 and ZEB2

**Figure 5:**
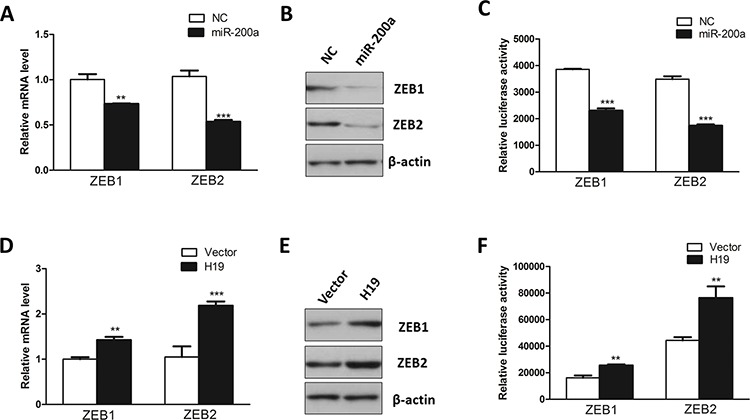
H19 functions as a decoy for ZEB1- and ZEB2-targeting miRNA **A.** MiR-200a was introduced into HT-29 cells. qRT-PCR was performed to assess the mRNA levels of ZEB1 and ZEB2. **B.** Western blot was conducted to evaluate the effect of miR-200a in regulating the protein levels of ZEB1 and ZEB2. **C.** Luciferase reporter assays were utilized to examine the interaction between miR-200a and ZEBs mRNA using reporters containing the 3′UTR region of ZEB1 or ZEB2. **D.** RT-PCR was used to monitor the effect of overexpression of H19 in the regulation of the mRNA levels of ZEB1 and ZEB2. **E.** Western blot was utilized to test the effect of overexpression of H19 in modulating the protein levels of ZEB1 and ZEB2. **F.** Luciferase reporter assay was performed to examine the effect of H19 in attenuating miR-200a-mediated suppression on the 3′UTR region of ZEB1 or ZEB2. **P < 0.01; ***P < 0.001.

To determine whether H19 regulates EMT by affecting miR-200a targets, we first evaluated the effect of miR-200a on its known targets ZEB1 and ZEB2 [43, 44]. ZEB1 and ZEB2, two well-known EMT-related transcription factors, bind to the corresponding elements E-boxes and subsequently suppress or activate gene transcription. Both ZEB1 and ZEB2 interact with E-boxes within promoter region of E-cadherin through their zinc finger domains, thereby repressing E-cadherin expression and other epithelial junction genes [6]. As previously reported [43, 44], enhanced expression of miR-200a dramatically suppressed ZEB1 and ZEB2 in both mRNA and protein levels (Figure 5A and 5B). On the other hand, we co-transfected miR-200a with ZEB1 or ZEB2 3′UTR-expressing luciferase reporters. Significant suppression on luciferase activity was observed after overexpression of miR-200a (Figure 5C). To verify whether H19 can rescue miR-200a-mediated inhibition of ZEB1 and ZEB2, we detected the mRNA and protein levels of ZEB1 and ZEB2 in H19-overexpressing HT-29 cells. As we expected, restored expression of H19 dramatically increased the mRNA and protein levels of ZEB1 and ZEB2 (Figure 5D and 5E). On the other hand, we utilized siRNA against H19 and examined the effect of H19 in modulating ZEB1 and ZEB2 expression. Consistent with previous study, downregulation of H19 resulted in reduction of ZEB1 and ZEB2 in both mRNA and protein levels (Supplementary Figure 3C and 3D). Furthermore, overexpression of H19 reversed endogenous miR-200a-mediated suppression on the luciferase reporters harboring 3′UTR from ZEB1 or ZEB2 genes (Figure 5F). These results suggest that H19 may interfere with miR-200a-mediated inhibition on ZEB1 and ZEB2, leading to the differentiation from epithelial cells towards mesenchymal cells.

### H19 promoted tumorigenesis *in vitro* and *in vivo*

**Figure 6:**
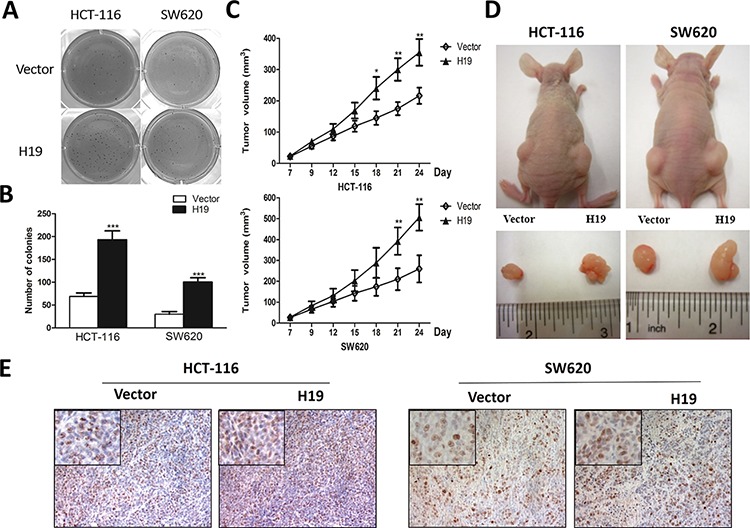
H19 promotes *in vitro* and *in vivo* tumor growth **A.** The capacity of anchorage-independent growth of HCT-116 and SW620 cells overexpressing H19 was assessed by soft agar assays. The captured photographs displayed the whole-well view of cell colonies after crystal violet staining. **B.** Images showed the colony formation capacity obtained from at least three independent experiments. **C.** The H19 and vector-transfected stable cells were subcutaneously injected into each side of nude mice for 4 weeks. Tumor volumes were measured and calculated. **D.** The representative dissected tumor tissues from nude mice were showed. **E.** The tumor tissues were subjected to Ki-67 staining. Representative pictures were captured and showed. *P < 0.05; **P < 0.01; ***P < 0.001.

To explore the biological importance of H19 in tumorigenesis, we examined the effect of H19 in regulating the capacity of colony formation in two colon cancer cells HCT-116 and SW620 because anchorage independent growth is an important characteristic for malignancy and EMT. Intriguingly, H19-overexpressing cells presented more and bigger colonies compared with control groups (Figure 6A and 6B). In order to further confirm the results from soft agar assay, an *in vivo* xenograft model was used. H19-overexpressing HCT-116 or SW620 cells and their parallel vector-carrying cells were subcutaneously injected into the same nude mice. Four weeks after injection, compared with control groups, H19-overexpressing cells displayed significant increase in the tumor size (Figure 6C and 6D). Thereafter, we also analyzed the expression patterns of E-cadherin and Vimentin in these tumor samples from nude mice. In accordance with our previous results, overexpression of H19 led to upregulation of Vimentin and downregulation of E-cadherin (Supplementary Figure 4). Furthermore, the cell proliferation marker Ki-67 was used for evaluating *in vivo* tumor growth and enhanced Ki-67 expression was observed in H19-overexpressing tumor tissue (Figure 6E), suggesting a potential oncogenic effect of H19.

### Expression of H19 was frequently increased in human CRC tissues

**Figure 7:**
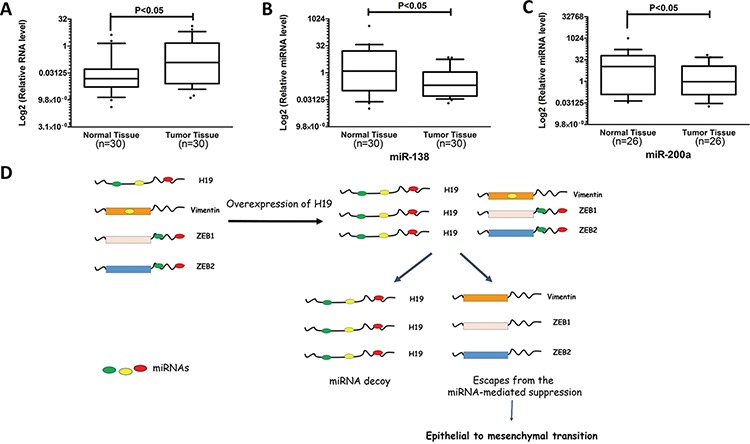
H19 is overexpressed in colon cancer patient specimens **A.** The expression levels of H19 in paired CRC and adjacent normal tissues were determined by qRT-PCR. **B.** Expression profiles of miR-138 in CRC and corresponding normal tissues were detected by qRT-PCR. **C.** Expression profiles of miR-200a in CRC and normal tissues were examined by qRT-PCR. **D.** Schematic overview of H19 regulatory network in epithelial to mesenchymal transition. LncRNA H19 shares miRNA-response elements with several EMT-related genes, namely Vimentin, ZEB1, and ZEB2. In mesenchymal cells, upregulated H19 acts as a miRNA sponge for several miRNAs that target these EMT-related genes. And increased H19 expression attenuates miRNA-mediated suppression on Vimentin, ZEB1, and ZEB2, leading to progression of epithelial to mesenchymal transition.

As previously described, H19 was frequently increased in colon cancer patients [12, 13]. We assessed the H19 expression level of in 30 paired CRC tissues and their corresponding adjacent normal colon tissues. In accordance with previous findings [12, 13], CRC samples displayed significant upregulation of H19 in mRNA level when compared with adjacent normal tissues (Figure 7A). We also examined the expression profile of miR-138 and miR-200a in 30 paired CRC samples. Consistent with previous studies, both of miR-138 and miR-200a presented significant downregulation in colon cancer samples when compared to the corresponding adjacent normal tissues (Figure 7B and 7C).

To further confirm the relationship between H19 expression and colon cancer progression, we compared the H19 expression level in colon cancer and normal tissues using public database Oncomine [46]. Six of nine data sets, which include microarray expression profiles classified into normal or colon cancer tissues, displayed higher H19 expression levels in colon cancer tissues rather than normal tissues (Supplementary Figure 5). These results indicate that increased H19 expression is a frequent event in human CRC tissues and H19 may involve in tumor progression of coloncancer. To summarize, a hypothetical model was depicted in Figure 7B

## DISCUSSION

In spite of encouraging advances in our knowledge of the molecular mechanisms of CRC, the prognosis for patients with advanced colon cancer remains unfavorable. Hence, it is of great importance to unravel the molecular switch controlling this malignant phenotype and elucidate the underlying mechanisms on the metastatic evolution of CRC. In particular, EMT is a powerful paradigm for studying the genetic progression of advanced stage solid tumor. Furthermore, in terms of that most of human malignancies arise from the epithelium tissues, the investigations on EMT will be beneficial to not only CRC but also the bulk of solid malignant tumors. Growing evidence elucidating the biochemical architecture on how EMT potentiates cell migration and metastasis have been discovered. However, nowadays the functional role of lncRNA and other RNA transcripts in modulating EMT is poorly understood. Despite the fact that approximately 90% of eukaryotic genome is transcribed [47], a surprisingly small portion of these RNA transcripts actually encodes functionally active proteins, indicating that the majority of RNA transcripts are non-protein-coding. Recently, long non-coding RNAs, which are more than 200 nucleotides in length, emerge as a new class of functional regulatory elements in modulating gene expression. A number of lncRNAs have been recently linked to diverse human diseases.

In the present study, we identified lncRNA H19 as a novel player in modulating EMT progress and revealed a previously unknown mechanism involving H19 and EMT-related miRNAs in cancer biology. Our finding that H19 promoted EMT revealed several important aspects. Herein, H19 was identified to be significantly upregulated in mesenchymal-like colon cancer cells. In accordance with this, stable overexpression of H19 remarkably induced epithelial cells to differentiate towards mesenchymal cells. Subsequent study demonstrated that H19 functioned as a miRNA decoy and abolished the endogenous suppressive effect on mesenchymal marker genes ZEB1, ZEB2, and Vimentin. Moreover, increased expression of H19 positively regulated *in vitro* colony formation as well as *in vivo* tumor growth. Finally, H19 was significantly upregulated in colorectal cancer patient tissues, indicating the pathological importance of H19 in tumor progression.

Consistent with our findings, a recent study by using *in vivo* crosslinking affinity purification experiment revealed that H19 directly interacted with RISC, where miRNAs recognize their complementary mRNA targets and exert their functions [41]. Further experiment verified that H19 associated with miRNA let-7 and modulated the expression of let-7 targets, which provides powerful evidence that H19 is a natural sponge for miRNAs. Moreover, based on the bioinformatics prediction combined with experimental analysis [41], H19 was found to associate with RISC and might contain various additional miRNA binding sites, indicating that H19 is likely to regulate other miRNAs except let-7. Aside from miR-138 and miR-200a, it seems interesting to investigate whether H19 might exert its function through titrating other unknown miRNAs.

According to the ceRNA hypothesis, lncRNAs communicated with other RNA transcripts throughmiRNA response elements as the letters of a novel RNA language [26]. In addition to lncRNAs, other RNA transcripts such as pseudogenes and circular RNAs have been demonstrated to serve as potential miRNA sponges. Without doubt, better bioinformatics algorithms and innovative molecular approaches will dramatically strengthen the understanding of the ceRNA hypothesis. For instance, the high-throughput sequencing of RNA isolated by crosslinking immunoprecipitation (HITS-CLIP) enables us to identify novel microRNA targets by decoding microRNA:mRNA interaction maps [48]. In addition, another newly developed technology termed crosslinking, ligation, and sequencing of hybrids (CLASH) provides an unbiased approach for the definition of putative miRNA targets and successfully identifies a high-confidence miRNA:mRNA interactome with a large dataset of high-confidence miRNA:mRNA interactions [49], which largely extend our knowledge of RNA interaction networks.

Our present study represents a paradigm that lncRNAs might serve as a ceRNA by sequestering miRNAs during EMT progress. A number of publications have showed that lncRNAs are powerful and vital *cis*- or *trans*-regulatory elements for gene expression [39, 50]. Unfortunately, in these serial recently established models, lncRNAs mainly function as scaffold guides to recruit protein partners and assist in modulating three-dimensional chromatin architecture. However, interestingly, our data herein indicate an innovative model to depict miRNA:lncRNA interaction during epithelial to mesenchymal transdifferentiation. And this novel mechanism will lead to a better understanding of epithelial to mesenchymal transition as well as other relevant human diseases.

## MATERIALS AND METHODS

### Cell lines

The human embryonic kidney 293 cells, colon cancer cell lines SW620 cells and HCT-116 cells were purchased from ATCC. HT-29 and HT-29 MTX resistant cells were kind gifts from Prof. Carlos J. Ciudad (University of Barcelona, Spain).

### Tissue specimens

Thirty paired primary colorectal cancer specimens and corresponding adjacent nontumor colon tissues were collected from tumor surgical resection in the Prince of Wales Hospital (The Chinese University of Hong Kong, Hong Kong, China). And all the human tissues were obtained with informed consent and this study was approved by the Clinical Research Ethics Committee at The Chinese University of Hong Kong.

### Cell transfection

Both the miRNA mimics and siRNAs were synthesized by Genepharma Company (Shanghai, China). Oligonucleotide and plasmid transfection were conducted by using the Lipofectamine™ 2000 transfection reagent (Invitrogen, USA), followed by the protocol recommended by the manufacturer. After 48 h transfection, the cells were collected and used for further investigations.

### Retrovirus transduction

H19 overexpression stable transfectants were constructed using retrovirus-mediated gene delivery method in human colorectal cancer cell lines. For generation of retrovirus, 2 μg expression vector pBABE-H19 or corresponding empty vector were co-transfected with equal amount of packaging vector pCL-Ampho into virus packaging HEK293 cells at 70% confluence in 100 mm culture dish using Lipofectamine 2000 reagent (Invitrogen, USA). After 24 hours post-transfection, the supernatant containing retrovirus were harvested and filtered by 0.45 μm pore size nitrocellulose membranes (Millipore, USA). The human colorectal cancer cell lines, namely HT-29, HCT-116 and SW620 cells, were infected with retrovirus particles together with 8 μg/mL Hexadimethrine bromide (Sigma-Aldrich, USA). After 48 hours post-infection, retrovirus infected cells were selected with puromycin (Sigma-Aldrich, USA) in the concentration of 0.7 μg/mL for HT-29 cells, 2 μg/mL for SW620 cells, and 0.9 μg/mL for HCT-116 cells. To maintain the stable expression of H19 in colorectal cancer cell lines, the infected cells were cultured in the selection medium with puromycin continuously. After antibiotics selection for around 2.5 weeks, the cells was harvested and the relative H19 RNA level was monitored by qRT-PCR.

### RNA extraction and real-time PCR

Total RNA was isolated by TRIZOL Reagent (Invitrogen, USA) following the manufacturer's instructions. After RNA extraction, RNA samples were reversely transcribed by High Capacity cDNA Reverse Transcription Kit (Applied Biosystems, USA). The Fast start Universal SYBR Green Master (Roche, USA) was applied for the quantitative RT-PCR. All the primer sequences were listed in Supplementary Table 1. The relative fold changes of candidate genes were analyzed by using 2^−ΔΔ^CT method.

### Western blot

Cell lysates were lysed by RIPA buffer (Sigma-Aldrich, USA) with Complete Protease Inhibitor Cocktail (Roche, USA). Cell lysates were transferred to 1.5 mL tube and kept at −20°C before use. SDS-PAGE was conducted to separate the cellular proteins. And all the cellular proteins within this study were separated by 5% stacking gel and 10% running gel. The molecular weight of candidate proteins were referred to the information of the Pre-stained SeeBlue rainbow marker (Invitrogen, USA) loaded in parallel. The membranes were probed with the following antibodies: E-cadherin (Cell Signaling Technology, USA), Vimentin (Santa Cruz, USA), ZEB1 (Santa Cruz, USA), ZEB2 (Zymed Laboratories, Invitrogen, USA), and β-actin (Sigma, USA). The blots were detected on Kodak film developer (Fujifilm, Japan).

### Colony formation assay

1 × 10^3^ cells H19-overexpressing stable cells and their corresponding vector control cells were seeded into 6 well plate. After culture for 12 days, cells were fixed with 70% ethanol and subsequently stained by 0.2% crystal violet solution. The images were captured and the colonies was counted by Image J software (National Institutes of Health, USA).

### Cell migration assay

Transwell migration assays were performed in a Boyden chamber with 8.0 μm pore (Corning, USA). 1 × 10^5^ H19-overexpressing HT-29 or SW620 stable cells and their corresponding vector control cells were seeded into the upper compartment. After the desired incubation period, the non-migrated cells on the upper chamber were removed. Afterwards, the cells were fixed with 4% paraformaldehyde for 20 minutes and stained with 0.2% crystal violet (Sigma-Aldrich, USA) for 10 minutes. Images were captured and the colony number was analyzed by Image J software (National Institutes of Health, USA).

### Soft agar assay

Both H19-overexpressing stable cells and their corresponding vector control cells were cultured in 100 mm culture dish. When the cells reaches 70% confluence, cells were trypsinized by 0.25% Trypsin (GirboBRL, USA) and then subjected to soft agar assay. Firstly, 6 well plates were pre-coated with 0.5% basal agar layer with culture media. Then, trypsinized cells were re-suspended in 0.35% upper agar layer and seeded into 6 well plate at the density of 1 × 10^3^cells per well. After culture for 10 days, cell colonies were visualized by 0.2% crystal violet staining. Images of stained cell colonies were captured and the colony number were counted by Image J software (National Institutes of Health, USA).

### Flow cytometry

H19-overexpressing stable cells and their corresponding vector control cells were washed with PBS and trypsinized by 0.25% Trypsin (GirboBRL, USA). After 2,000 rpm/min centrifugation for 2 minutes, the supernatant was removed and the cell pellets were re-suspended and fixed in 70% ethanol at 4°C overnight, Afterwards, the cells were stained with staining buffer (50 μg Propidium iodide/ml, 0.37 mg/ml EDTA, 50 μg RNAse/ml, 1% Triton X-100) at 4°C for 20 min. After the Propidium iodide staining, the cells were subjected to cell cycle analysis using LSR Fortessa (BD Biosciences, USA) and FACSDiva software (BD Biosciences, USA).

### RNA immunoprecipitation

HEK293 cells were rinsed with cold PBS and fixed by 1% formaldehyde for 10 minutes. After centrifugation, cell pellets were collected and then re-suspended in the NP-40 lysis buffer supplemented with 1mM PMSF, 1 mM DTT, 1% Protease Inhibitor Cocktail (Sigma-Aldrich, USA) plus 200 U/ml RNase Inhibitor (Life Technologies, USA). The cell lysates were stored at −80°C before use. The supernatant from cell lysates was collected by high-speed centrifugation. To generate antibody-coated beads, Protein G Sepharose 4 Fast Flow bead slurry (GE Healthcare, USA) was rinsed with NT2 buffer (50 mM Tris-HCl, 150 mM NaCl, 1 mM MgCl_2_, 0.5% NP-40) and then incubated with antibody against Ago2 (Abcam, UK). The mouse IgG (Sigma-Aldrich, USA) was used as negative control. For RNA immunoprecipitation, the supernatant was incubated with the antibody-coated Sepharose beads overnight. The beads were rinsed with cold NT2 buffer, followed by incubation with 10 mg/ml proteinase K (Sigma-Aldrich, USA). The RNA bound to Ago2 antibody was extracted with TRIzol reagent (Invitrogen, USA).

### Luciferase reporter assay

By using of the Lipofectamine™ 2000 transfection reagent, HEK293 cells cultured in 24 well plate were co-transfected with luciferase reporter plasmids and miRNA mimics as well as the internal control pRSV-β-Galactosidase vector. After 48 hour post-transfection, HEK293 cells were lysed by lysis buffer (25 mM Tris-phosphate, 1% Triton X-100, 1 mM DTT, 2 mM EDTA, 10% Glycerol, pH = 27.8). After centrifugation at 14,000 rpm for 3 minutes, the supernatant was transferred to a new 1.5mL tube. The luciferase activity was monitored by mixing 50 μL supernatant with 50 μl luciferase assay buffer (265 μM ATP, 2.70 mM MgSO_4_, 1.07 mM MgCl_2_, 135 μM Coenzyme A, 20 mM Tricine, 0.1 mM EDTA, 33.3 mM DTT, 235 μM D-Luciferin) by using the Gloxmax 20/20 Luminometer (Promega). The β-Galactosidase activity from the pRSV-β-Galactosidase vector was used for the normalization of the luminescence levels. O-nitrophenyl-β-galactoside (ONPG) colorimetric assays were performed to measure the β-Galactosidase activity. 50 μl supernatant from aforementioned cell extract was mixed with 100 μl of ONPG solution (0.666 mg/ml ONPG, 40 mM NaH_2_PO_4_, 60 mM Na_2_HPO_4_, 10 mM KCl, 1 mM MgSO_4_, 2% β-mercaptoethanol). The β-Galactosidase activity was evaluated by the measurement of o-nitrophenol by using the ELISA plate reader (Bio-Rad, USA) at the wavelength of 415 nm.

### Xenograft tumor model

Both H19-overexpressing HCT-116 or SW620 cells and their parallel control cells were grown in 100 mm culture dish. When the cultured colon cancer cells reach 80% confluence, cells were trypsinized and collected. 1 × 10^6^ H19-overexpressing HCT-116 or SW620 cells, and their parallel control cells were subcutaneously implanted into the posterior flank of the same nude BALB/c mice (*n* = 4) aged 4 weeks. The tumor cells were allowed to grow for 4 week. The tumor growth was evaluated by measurement of the length and the width with electronic calipers and the tumor volume was calculated using the formula: Volume = (Length × Width^2^)/2. One month later, the nude mice were sacrificed and the tumor tissues were excised and fixed in 4% Paraformaldehyde solution for further study. The tumor growth was evaluated by the value of tumor volume (mean ± SD), which was plotted against time. Animal handling was conducted in accordance with the institutional LASEC Guidelines in The Chinese University of Hong Kong.

### Immunohistochemistry

Tumor specimens from nude mice were fixed in 4% paraformaldehyde and then embedded in paraffin. Sections were used for the analysis of Ki-67 expression by Ki-67 antibody (Calbiochem, USA). Ki-67 expression was visualized by using the 3, 3-diaminobenzidine substrate.

### Statistics

Experimental results are presented as Mean ± S.E.M. Comparisons between two groups were conducted using two-tail Student's *T*-test and differences were considered to be statistically significant when *P* value is less than 0.05.

## SUPPLEMENTARY FIGURES, AND TABLE


