# Supplementary material for: The lncRNA H19 promotes epithelial to mesenchymal transition by functioning as miRNA sponges in colorectal cancer
Source: Oncotarget. 2015 Jun 5;6(26):22513–25. doi: 10.18632/oncotarget.4154 (PMC4673179; doi:10.18632/oncotarget.4154)
Supplement: Supplementary file 1 [file oncotarget-06-22513-s001.pdf]

SUPPLEMENTARY FIGURES, AND TABLE

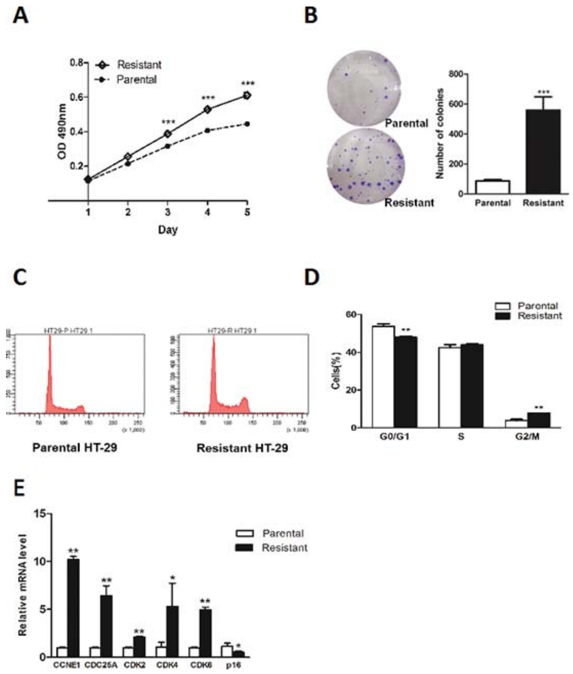

Supplementary Figure S1: Methotrexate resistant HT-29 cells displayed enhanced cell growth. **A.** Equal numbers of parental and resistant cells were seeded into 96 well plate. Methotrexate resistant HT-29 cells displayed increased cell proliferation. **B.** 1×10^3 parental and resistant HT-29 cells were seeded into 6 well plate and cultured for 12 days. The cell colonies were visualized by crystal violet and counted. Increased numbers of colonies were found in resistant cells. **C&D.** Flow cytometry was utilized to evaluate the difference in cell cycle progression between parental and resistant cells. Decreased G0/G1 phase and increase G2/M phase were observed in resistant cells. **E.** Several G1/S phase transition marker genes were monitored by RT-PCR. *$P < 0.05$; **$P < 0.01$; ***$P < 0.001$.

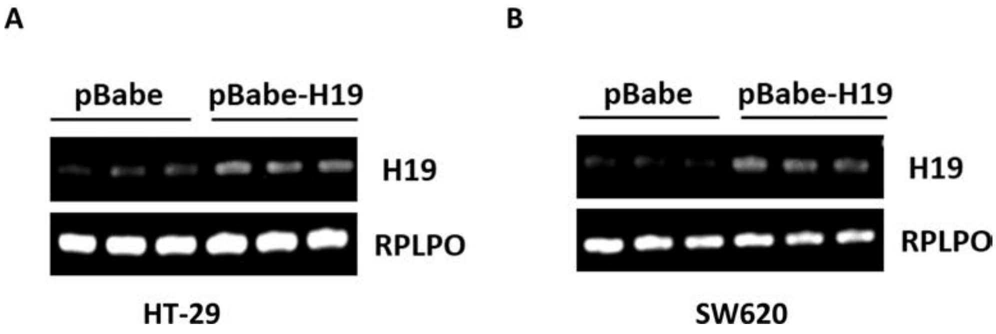

Supplementary Figure S2: Relative RNA level of H19 was monitored by semi-quantitative RT-PCR. Upregulation of H19 was observed in both HT-29 cells **A.** and SW620 cells **B.**

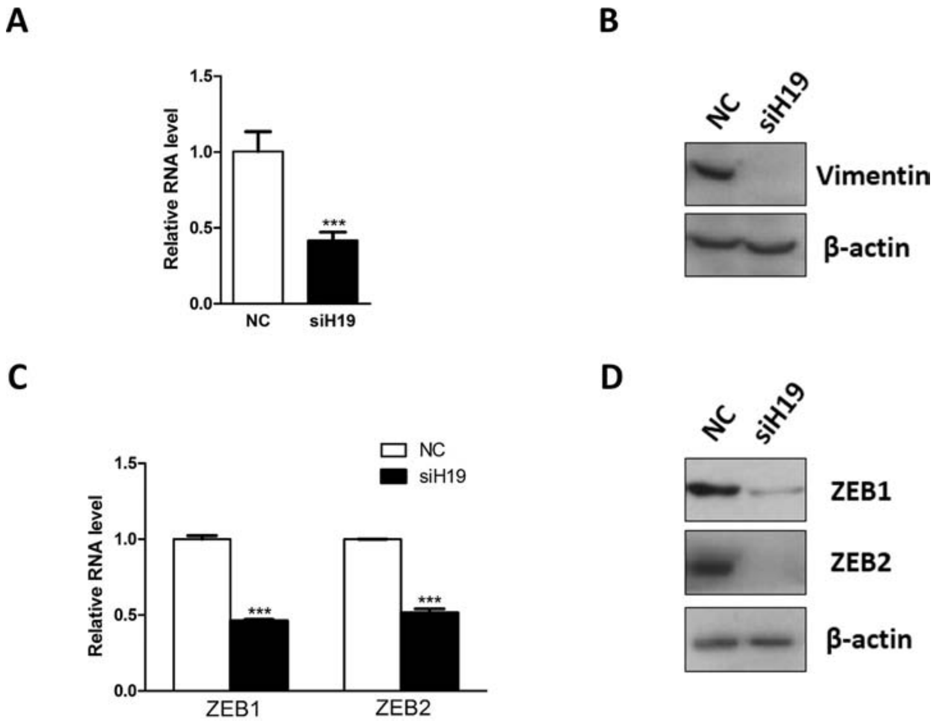

Supplementary Figure S3: The expression patterns of Vimentin, ZEB1 and ZEB2 were examined after downregulation of H19 by siRNA. Reduction of H19 triggered downregulation of Vimentin **A&B.** ZEB1 and ZEB2 **C&D.**

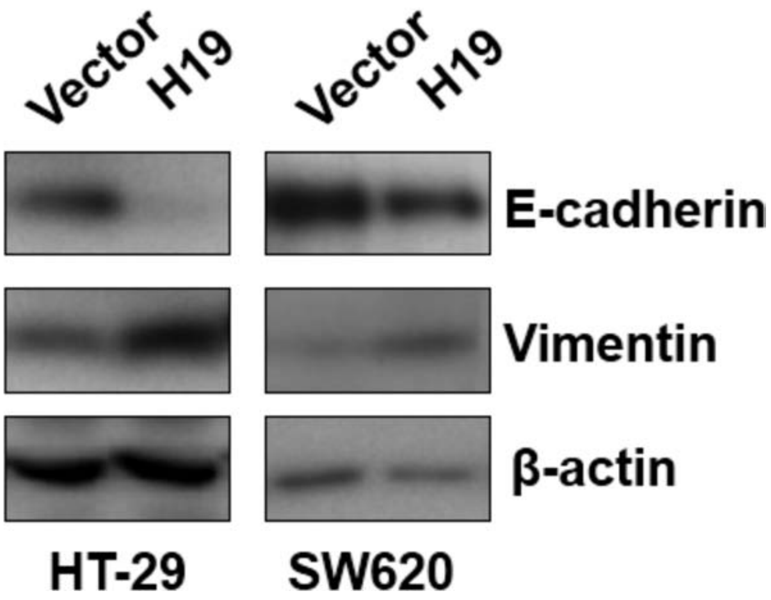

Supplementary Figure S4 The expression profiles of E-cadherin and Vimentin in tumor samples were examined. Stable expression of H19 increased the expression of Vimentin and decreased the expression of E-cadherin.

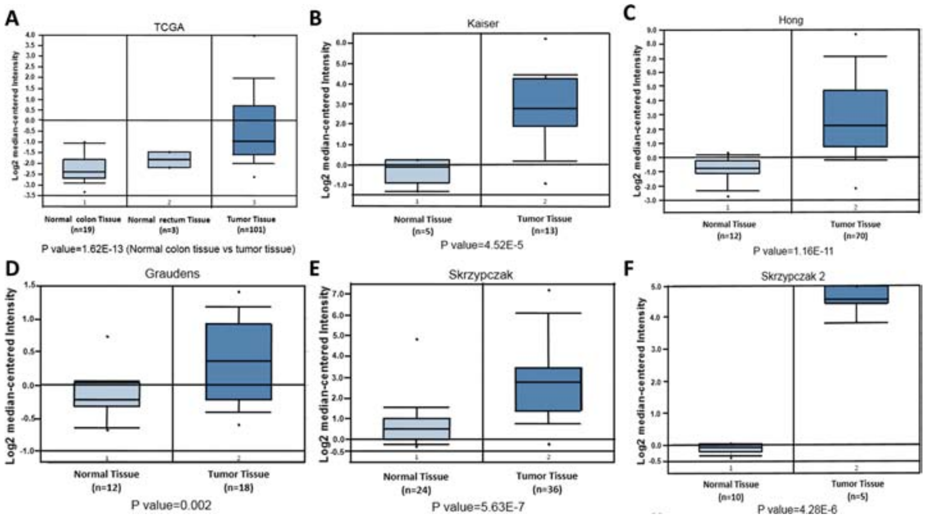

Supplementary Figure S5: Expression profile of H19 in CRC specimens from online public database Oncomine. A–F. Increased expression of H19 in tumor tissues was identified by six independent studies on human colorectal cancers. Data and statistics analysis were obtained from website http://www.oncomine.org.

Supplementary Table S1. Primer sequences used in this study.

Name	Primer sequences used for plasmid construction
H19-luc-F	AGCAGTTTAAACGGGAGGGGGTGGGATGGGT
H19-luc-R	GCTCTAGAGCTGTAACAGTGTTTATTG
pBabeH19-F	CGCGGATCCGGGAGGGGGTGGGATGGGT
pBabeH19-R	ACGCGTCGACGCTGTAACAGTGTTTATTG
VIM-CDS-luc-F	AGCAGTTTAAACCCTCCTACCGCAGGATGTT
VIM-CDS-luc-R	GCTCTAGAGATGTCCTCGGCCAGGTT
**Name**	**Primer sequences used for RT-PCR**
CUDR_F	GCACCCTAGACCCGAAA
CUDR_R	GCCACCTGGACGGATAT
TINCR_F	TGTGGCCCAAACTCAGGGATACAT
TINCR_R	AGATGACAGTGGCTGGAGTTGTCA
ST8SIA3_F	AGGAAGCCTGAGAGTTGGC
ST8SIA3_R	CTCAGTGGGGAAGACTCCAG
Linc_MD1_F	CACTGCCAGCTCTGGAAAAT
Linc_MD1_R	ACTTGGTTCCGTTTGACCAG
H19-F	TGCTGCACTTTACAACCACTG
H19-R	ATGGTGTCTTTGATGTTGGGC
RPLPO_F	CCGGATATGAGGCAGCAGTT
RPLPO_R	GAAGGCTGTGGTGCTGATGG
Vimentin-F	GACGCCATCAACACCGAGTT
Vimentin-R	CTTTGTCGTTGGTTAGCTGGT
E-cadherin_F	CGAGAGCTACACGTTCACGG
E-cadherin_R	GGGTGTCGAGGGAAAAATAGG
ZEB1_F	ATCATCGCTACTCCTACTG
ZEB1_R	TCTTCCCTTGTCAAACTC
ZEB2_F	CAAGAGGCGCAAACAAGCC
ZEB2_R	GGTTGGCAATACCGTCATCC
CDK2_F	ATGGAGAACTTCCAAAAGGTGGA
CDK2_R	CAGGCGGATTTTCTTAAGCG
CDK4_F	TTGCATCGTTCACCGAGATC
CDK4_R	CTGGTAGCTGTAGATTCTGGCCA
CDK6_F	TGCACAGTGTCACGAACAGA
CDK6_R	ACCTCGGAGAAGCTGAAACA

(*Continued*)

Cyclin E1_F	CAGATTGCAGAGCTGTTGGA
Cyclin E1_R	TCCCCGTCTCCCTTATAACC
p16_F	ATGGAGCCTTCGGCTGACT
p16_R	GTAACTATTCGGTGCGTTGGG
CDC25A_F	ACAGCTCCTCTCGTCATGAGAAC
CDC25A_R	GGTCTCTTCAACACTGACCGAGT
DHFR_F	ATCGGCAAGAACGGGGA
DHFR_R	TCTGGAAAGAAAATGAGC
U6	CTCGCTTCGGCAGCACA
miR-200a-3p	TAACACTGTCTGGTAACGATGT
miR-138-5p	AGCTGGTGTTGTGAATCAGGCCG
